# FcεR_1_-Mediated Mast Cell Reactivity Is Amplified through Prolonged Toll-Like Receptor-Ligand Treatment

**DOI:** 10.1371/journal.pone.0043547

**Published:** 2012-08-16

**Authors:** Rohit Saluja, Ingrid Delin, Gunnar P. Nilsson, Mikael Adner

**Affiliations:** 1 Clinical Immunology and Allergy Unit, Department of Medicine, Karolinska Institutet, Stockholm, Sweden; 2 Experimental Asthma and Allergy Research, The Institute of Environmental Medicine, Karolinska Institutet, Stockholm, Sweden; 3 The Centre for Allergy Research, Karolinska Institutet, Stockholm, Sweden; University of Cambridge, United Kingdom

## Abstract

**Background:**

Mast cell-derived mediators mediate several of the pathological features of asthma. Microbial infections induce asthma exacerbations in which the contribution of mast cells remains incomprehensible.

**Principal Findings:**

In this study we have investigated the characteristic expression pattern of Toll-like receptors (TLRs) 1–9 and the effect of TLR ligand treatment on IgE-receptor mediated mast cell reactivity. For the studies we employed *in vitro* differentiated connective tissue like mast cells (CTLMC) and mucosal like mast cells (MLMC) from mice. Both phenotypes were treated for 24 h or 96 h with ligands for TLR1/2, TLR2/6, TLR3 and TLR4, before activation with IgE and antigen. Prolonged exposure (96 h) with TLR-ligands promoted mast cell reactivity following IgE-receptor activation. TLR4 activation with LPS generated the most pronounced effect, with an enhanced degranulation and secretion of leukotrienes, cytokines and chemokines, in both CTLMC and MLMC. The effect of LPS was mediated through a Myd88-dependent pathway and the increased effect involved JNK-dependent pathway.

**Conclusion:**

We find that prolonged exposure of mast cells to pathogens/TLR-ligands modulates their effector responses by priming them for increased release of several inflammatory mediators when subsequently activated by IgE-receptors. These data suggest that infections might exaggerate the severity of allergic reactions such as in asthma, by enhancing mediator release from mast cells.

## Introduction

Asthma is a complex clinical syndrome with airway hyperresponsiveness, tissue remodeling and chronic airway inflammation as the major characteristics. It is today well recognized that mast cell activation and concomitant release of mast cell mediators contribute to these pathological features [1]. Inhibition of histamine and cysteinyl leukotrienes (CysLTs) diminishes most of the allergen-induced early and late phase asthma reactions [2]. In addition to histamine and CysLTs, other mast cell-derived mediators as proteases, prostaglandins and other eicosanoids, cytokines, chemokines and growth factors might promote different phases of the pathology, such as remodeling and inflammation [1], [3]–[5].

For patients with asthma, exacerbations are a major cause of morbidity. Most commonly the acute exacerbations are associated with viral infections [6], but also bacterial infections have been implicated as a direct trigger [7] or subsequent to the viral infection [8]. The mechanisms for asthma exacerbations upon infections are not clear. However, clinical evidence supports an interaction between allergy (IgE-mediated) and viral infection in the cause of exacerbations [9], [10]. An experimental study demonstrated that inoculation of human rhinovirus potentiated histamine release upon allergen provocation in allergic, but not in non-allergic subjects [11], further suggesting an effect on IgE-mediated mast cell reactivity. Most likely the infection affects both epithelial and smooth muscle cells, as well as inflammatory cells, and promotes the airway hyperresponsiveness. Since mast cell mediators are tightly connected to asthma, both airway obstruction and inflammation, one reasonable speculation would be that mast cell reactivity is increased upon infection.

Mast cells express a number of different pattern-recognition receptors, including Toll-like receptors (TLRs) [12]. TLR-ligands can directly activate mast cells causing release of mast cell mediators in a distinct pattern [13]–[16]. Whether TLR-ligands affect IgE-mediated reactivity, as suggested by the experimental *in vivo* studies [11], is not clear [17]. There are conflicting reports demonstrating that co-treatment with TLR-ligands and aggregation of FcεRI either suppress degranulation and leukotriene synthesis [18], [19], down-regulates FcεRI expression [20], or increase cytokine release without affecting degranulation or synthesis of eicosanoids [21].

To further investigate if infectious stimulation affects mast cell reactivity we have in this study, in contrast to previous studies, used a prolonged exposure protocol where mast cells have been treated with TLR ligands for 24 h or 96 h before the cells were activated by aggregation of FcεRI. We investigated the effect of TLR 1/2, TLR 2/6, TLR 3 and TLR 4 on FcεRI-mediated degranulation, leukotriene synthesis, and release of cytokines and chemokines. In addition, this study has investigated differences in the functional response of *in vitro*-developed connective tissue like mast cells (CTLMC) and mucosal like mast cells (MLMC), to IgE-receptor activation and the effect that TLR agonists might exert on this response.

## Results

### Expression of TLRs on CTLMC and MLMC

**Figure 1:**
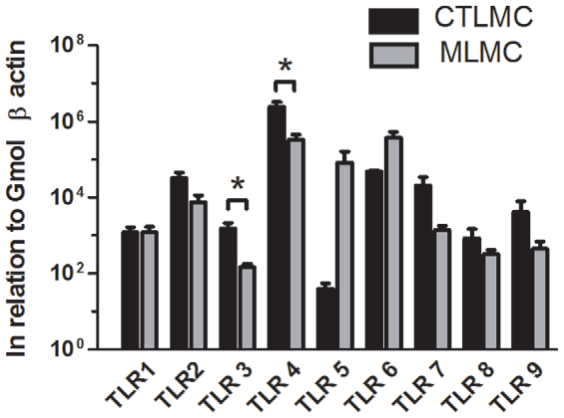
Relative expression of TLRs (1–9) in CTLMC and MLMC. RNA purified from CTLMC and MLMC was subjected to quantitative real-time PCR using SYBR Green for the indicated TLRs. The relative gene expression levels were determined in triplicates by difference in Ct numbers and results were described as in relation to Gmol β actin. Data are plotted as mean ± SEM and derived from 4–6 independent experiments.

We first investigated the basal mRNA expression of TLRs. Real time PCR analysis indicated expression of all TLRs (Fig. 1). There was a tendency for higher expression of TLR2, TLR4 and TLR6 in both phenotypes and TLR3 and TLR4 were significantly higher expressed in the CTLMC than MLMC. On the basis of these results we selected LPS as an activator of TLR4, FSL-1 as an activator of TLR2/6, and PamOct2C-(VPGVG) 4VPGKG (POC) as an activator of TLR1/2 to discriminate between these two receptor hetero-dimers. In addition we included poly(I:C) as an activator of TLR3, representing viral activation.

### Prolonged Exposure to TLR Ligands Increase Antigen-mediated Mast Cell Degranulation

**Figure 2:**
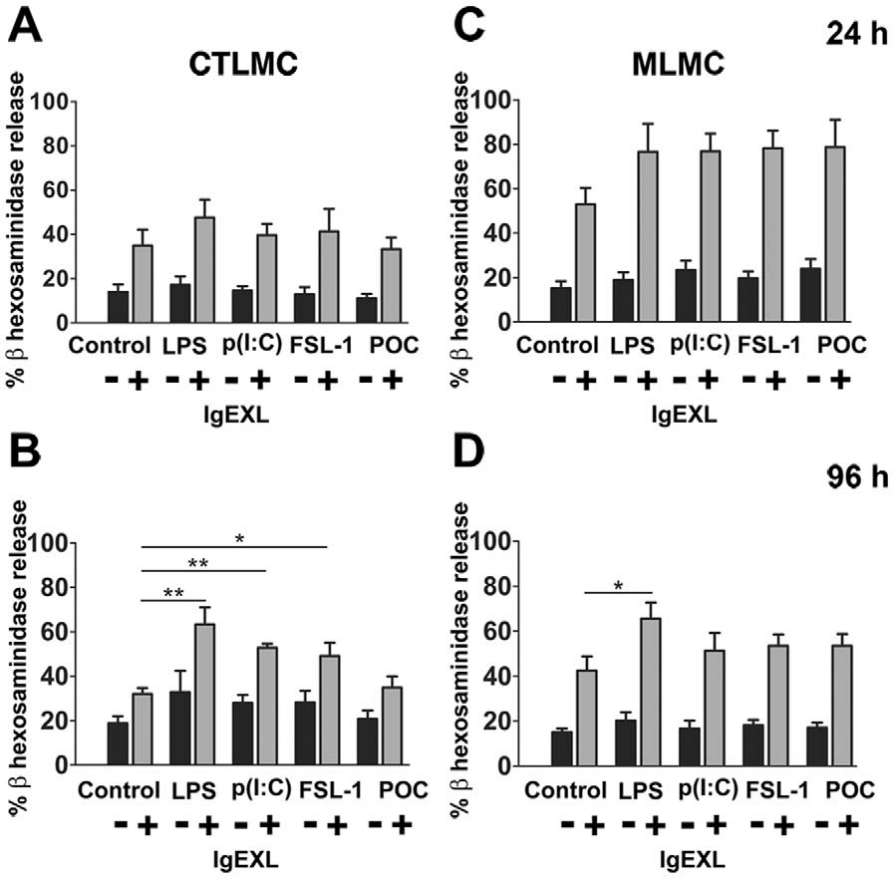
Pre-treatment with TLR agonists increase FcεRI-mediated degranulation in CTLMC and MLMC. Cells were treated with different TLRs agonists for 24 h (*A* and *C*) or 96 h (*B* and *D*) and were sensitized with IgE followed by antigen. Degranulation was determined by measuring the release of β-hexosaminidase in duplicates. The release was calculated as a percentage of total β-hexosaminidase content released following activation. Results are represented as the mean ±SEM for 5 independent experiments. *p<0.05, **p<0.01 significantly different from the respective controls.

Previous studies on the effect of TLR-ligands on IgE-mediated mast cell reactivity used short exposure protocols or simultaneous addition of TLR-ligands and antigen to cross-link FcεRI [17]. We could confirm earlier presented data [18]–[21] that simultaneous treatment of IgE sensitized MLMC and CTLMC with TLR ligands and antigen did not cause further increase in degranulation or leukotriene secretion as compared to antigen alone (data not shown). We next investigated the influence of prolonged treatment with TLR-ligands and compared the effect of cells treated for 24 and 96 h. By themselves, none of the TLR agonists caused in neither CTLMCs nor MLMCs treated for 24 h (Fig. 2A and 2C) or 96 h (Fig. 2B and 2D). When we further explored the influence of LPS, poly(I:C), FSL-1 and POC, we found that 24 h did not exhibit any significant effect on the subsequent antigen-induced mast cell degranulation, but a trend of increased degranulation (Fig. 2A and 2C) could be observed. However, when CTLMCs were treated for 96 h, LPS, poly(I:C) and FSL-1 enhanced antigen-mediated mast cell degranulation significantly. In CTLMC the degranulation upon IgE-receptor aggregation was 32±6% for untreated cells (Fig. 2B), while pre-treatment induced significant increase in degranulation: LPS 63±7% (p<0.01), poly(I:C) 52±2% (p<0.01), and FSL-1 49±6% (p<0.05). Similar results were obtained in MLMC after LPS exposure: untreated 42±6% and LPS-treated 65±7% (p<0.05; Fig. 2D). These results suggest that LPS or other TLR agonists can prime the cells for enhanced mediator release when subsequently triggered by IgE receptor cross linking.

### Increase in CysLT and LTB_4_ Secretion by CTLMC and MLMC after 96 h Treatment with TLR Agonist

**Figure 3:**
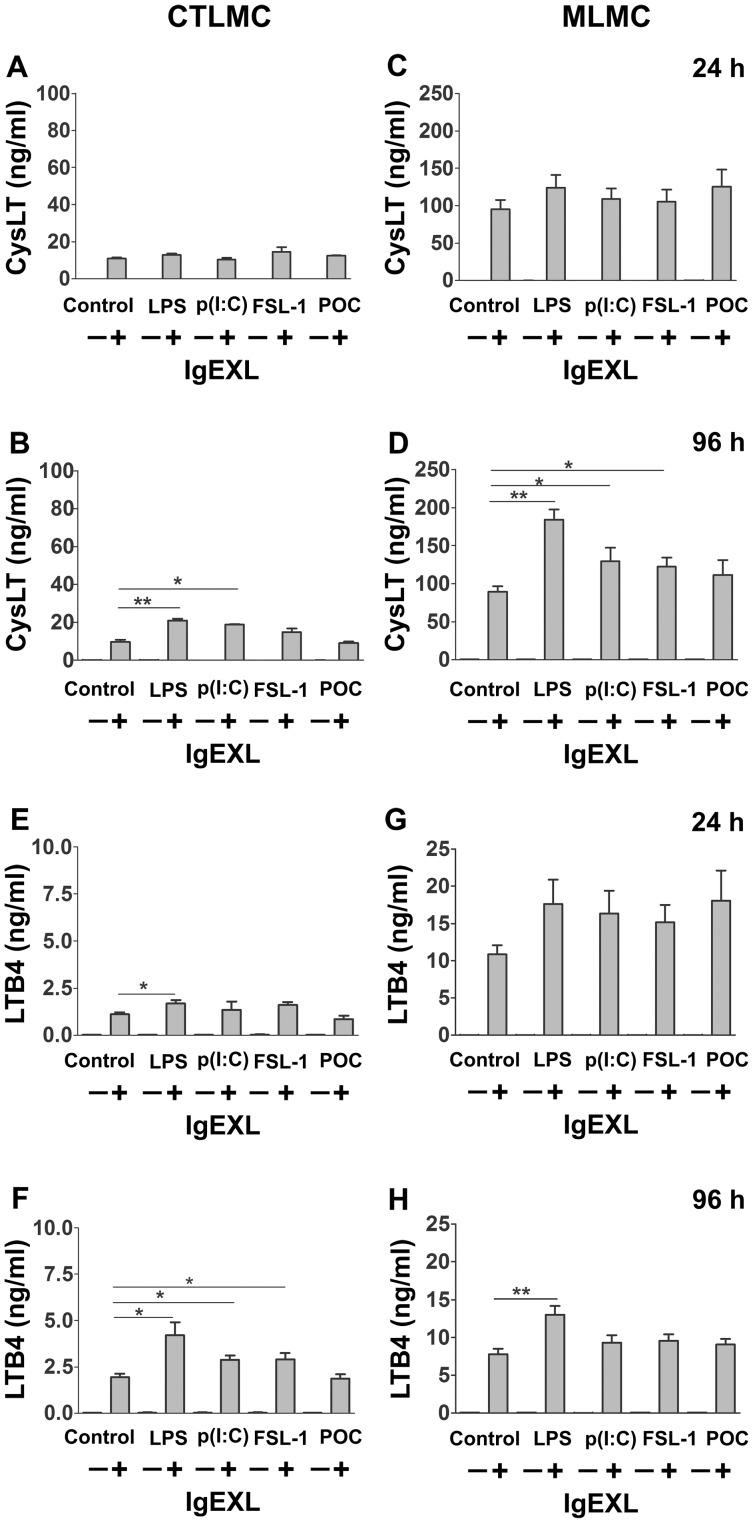
Pre-treatment with TLR agonists increase leukotriene products in CTLMC and MLMC. Cells were treated with different TLRs agonists for 24 h (*A*, *C*, *E*, *G*) and 96 h (*B*, *D*, *F*, *H*), CTMLC (*A*, *B*, *E*, *F*) and MLMC (*C*, *D*, *G*, *H*) were sensitized with IgE by antigen. CysLT and LTB_4_ were measured in the culture supernatant by ELISA in duplicates. Results are represented as the mean ±SEM for 5 independent experiments. *p<0.05, **p<0.01 significantly different from the control cells without IgE receptor cross-linking.

Since TLRs caused an enhanced IgE-mediated β-hexosaminidase release in both CTLMCs and MLMCs, we next determined whether 24 or 96 h treatments with TLR ligands also affect CysLT and LTB_4_ production. In untreated cells, an increase in CysLTs and LTB_4_ was found following IgE-receptor cross-linking in both cell populations and at both time-points (Fig. 3). In contrast, limited secretion was detected from cells treated with TLR-agonists for 24 or 96 h without IgE-receptor activation.

The 24 h treatment with TLR agonists did not show any increase in antigen-mediated secretion of CysLT (Fig. 3A and 3C). In contrast, 96 h treatment with LPS and poly(I:C) induced an amplified secretion of CysLTs in CTLMCs (20.9±1.1 and 18.7±0.3, respectively, vs. 9.7±1.0 without treatment; Fig. 3B), while 96 h treatment with LPS, poly(I:C) and FSL-1 induced an increase in secretion of CysLTs in MLMCs (184±13, 130±18 and 123±12 ng/ml, respectively, vs. 89±7 ng/ml for without treatment; Fig. 3D).

For the secretion of LTB_4_, the only significant effect induced by a 24 h pre-treatment with TLR agonists was shown by LPS mediated enhancement in CTLMC (1.7±0.2 vs. 1.1±0.1 ng/ml without treatment; Fig. 3E). 96 h prolonged exposure to LPS, poly(I:C) or FSL-1 significantly augmented the secretion by CTLMC (4.2±0.7, 2.9±0.2 and 2.9±0.3, respectively, vs. 1.9±0.2 without treatment; Fig. 3F). LPS had the same effect on MLMC (13.0±1.1 vs. 7.8±0.7 ng/ml without treatment; p<0.01; Fig. 3H).

### Effect of TLR Agonists on IgE-mediated Pro-inflammatory Cytokine Release in CTLMC and MLMC

**Figure 4:**
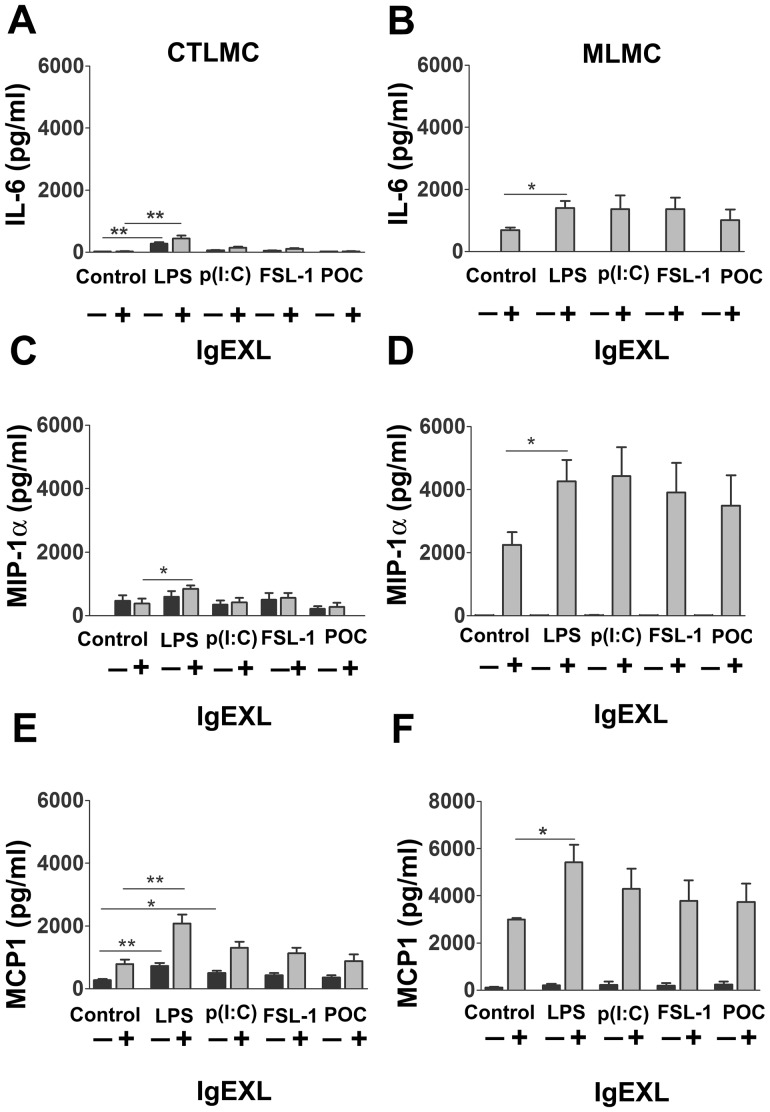
IgE-receptor induced pro-inflammatory cytokine and chemokine production by mast cells are enhanced upon pre-treatment with TLRs agonists. CTLMC (*A*,*C*,*E*) and MLMC (*B*,*D*,*F*) were treated for 96 h with different TLR agonists and then sensitized with IgE followed by antigen for 6 h. Culture supernatants were analysed for the presence of IL-6 (*A*,*B*), MIP-1α (*C*,*D*) and MCP-1 (E,F) by Luminex in duplicates. Data are mean ± SEM and representative of 5 independent experiments. *p<0.05, **p<0.01 significantly different from the respective controls.

To investigate the effect of prolonged exposure to TLR agonists on IgE-mediated release of cytokines, we measured the release of IL-1β, IL-10, IL-13, IL-17, TNF-α, IFN-γ, IL-6, MCP-1, MIP-1α, GM-CSF. Cells were cultured for 96 h with or without TLR agonist, washed, replated in fresh medium and cultured for an additional 6 h where after supernatants were harvested for cytokine analysis. We found that the release of IL-6, MIP-1α and MCP-1 were augmented by LPS, and to some extent by poly(I:C), in both CTLMC and MLMC (Fig. 4). For these cytokines, IgE-receptor cross-linking caused a strong release especially in MLMC. In CTLMC, LPS alone induced a release of IL-6 and MCP-1, both at baseline conditions and after IgE-receptor cross-linking (p<0.01; Fig. 4A and 4E); and a release of MIP-1α after IgE-receptor cross-linking (p<0.05; Fig. 4C). Furthermore, poly(I:C) induced release of MCP-1 (p<0.05; Fig. 4E). In MLMC, IgE-receptor aggregation caused release of IL-6 (Fig. 4B), MIP-1α (Fig. 4D) and MCP-1 (Fig. 4F). Prolonged treatment of MLMC with LPS caused a further enhancement in the release of these cytokines and chemokines.

### TLR-agonists Enhance Mast Cell Reactivity through a MyD88 Dependent Pathway

**Figure 5:**
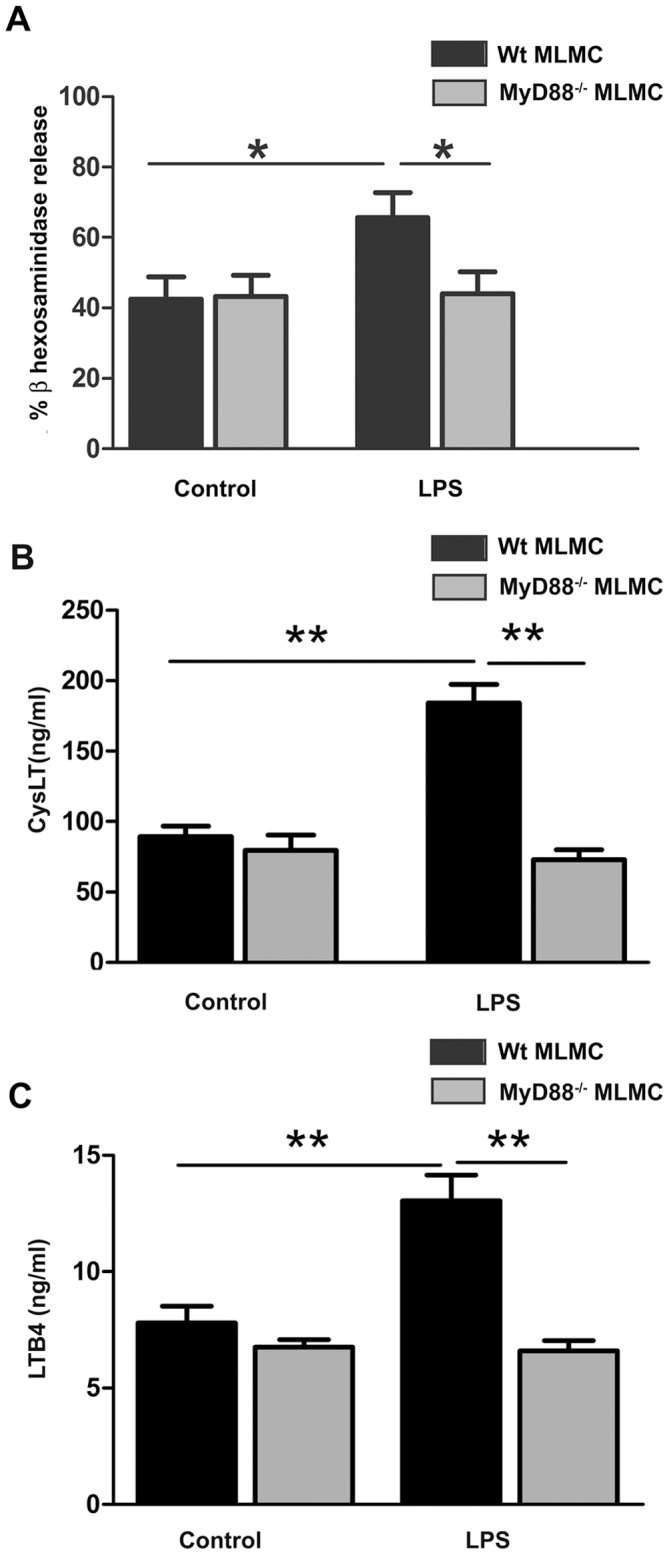
Pre-treatment with LPS did not increase FcεRI-mediated degranulation and leukotrienes release in MyD88−/− MLMC. MLMCs were treated with different LPS for 96 h and were sensitized with IgE followed by antigen. Degranulation was determined by measuring the release of β-hexosaminidase in duplicates. The release was calculated as a percentage of total β-hexosaminidase content released following activation (A). CysLT and LTB_4_ also exhibited the similar pattern as measured in the culture supernatant by ELISA (B and C). Results are represented as the mean ±SEM for 5 independent experiments. *p<0.05, **p<0.01 significantly different from the respective controls.

Since TLR4 signals through two main pathways; MyD88-dependent and -independent pathways [22], we investigated which of pathways that contributes to the observed long-term effects (96 h) in the mast cells. In this set of experiments we used MLMCs since they exhibited higher extent of mediator release as compared to CTLMCs. As expected, MLMCs from MyD88^−/−^ mice, did not exhibit LPS-mediated amplification of FcεRI-mediated degranulation (Fig. 5A). Similiar results were observed in case of CysLT and LTB_4_ release, where 96 h treatment with LPS did not induce an increase in secretion of CysLTs and LTB_4_ in MLMCs from MyD88^−/−^ mice (Fig. 5B and 5C).

To investigate the effect of prolonged exposure to TLR agonists on IgE receptor expression, we measured FcεR1 in MLMC by flow cytometer. The 96 h pre-treatment with different TLR agonists did not show any effect on FcεR1 expression. To examine if pre-treatment of the used TLRs agonist can influence the TLRs expression, mRNA for TLR1–TLR9 was analysed by Real Time PCR. However, no increase of TLRs expression was obtained following treatment with the TLR agonists (data not shown).

### The LPS-mediated Enhancement of Mast Cell Reactivity is Mediated through JNK

To investigate the effect of prolonged exposure to TLR agonists on IgE receptor expression, we measured FcεR1 in MLMC by flow cytometer. The 96 h pre-treatment with different TLR agonists did not show any effect on FcεR1. Neither could we observe any effect on TLR mRNA expression (data not shown).

**Figure 6:**
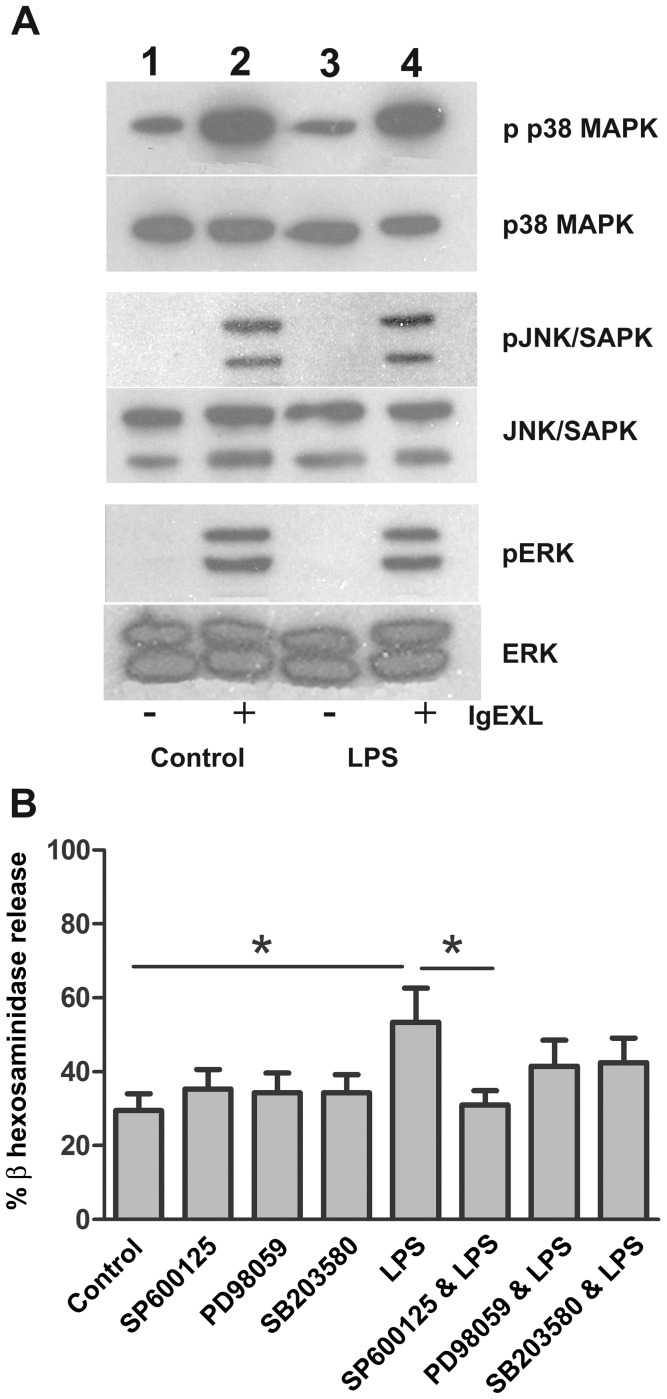
MAPK activation induced by LPS or IgE cross-linking. (*A*) MLMC were treated with LPS (1 µg/ml) for 96 h, IgE cross-linking (IgE-XL) or combination with LPS and IgE-XL (10 min). Phosphorylation of ERK, p38 MAPK and JNK was detected by phosphospecific antibodies against the respective kinases by Western blot with reference to the total kinases present in whole cell lysate. *Lane 1*: Control resting cells; *Lane 2*: control cells with IgE-XL; *Lane 3*: LPS treated cells; *Lane 4*: LPS treated cells exposed to IgE-XL (*B*) To explore the role of MAPKs involved in LPS triggered induction of degranulation from activated mast cells following IgE-XL, MLMC were preincubated with JNK inhibitor SP 600125 (1 µM), ERK inhibitor PD 98059 (1 µM) and p38 MAPK inhibitor SB 203580 (10 µM) in presence and absence of LPS (1 µg/ml) for 96 h. The cells were then sensitized challenged with IgE for 90 min and further treated with antigen for 30 min. Supernatants were collected in duplicates to evaluate β-hexosaminidase activity. Release was calculated as a percentage of total β-hexosaminidase. Results are presented as the mean ± SEM for 5 independent experiments. **p<0.01.

We next investigated the effect of LPS treatment on the MAPK pathways, including ERK, JNK, and p38 MAP kinase. As revealed by their phosphorylation pattern, all three kinases were activated following IgE-receptor activation (Fig. 6A). Phophorylation of the MAP-kinases was not affected by LPS treatment for 96 h, compared to untreated controls (Fig. 6A). No significant effect of LPS on IgE-receptor mediated phosphorylation of the investigated kinases was detected. To further investigate the involvement of MAPKs on the LPS-mediated enhancement we used pharmacological inhibitors against JNK, p38 MAPK and ERK. We first measured the toxicity of the drugs on mast cells during prolonged culture, and choose concentrations that did not affect mast cell viability: JNK (SP600125, 1 µM), p38 MAPK (SB203580, 10 µM) and ERK (PD98059, 1 µM). MLMC were treated with LPS ± inhibitors for 96 h, after which the cells were activated by IgE and antigen. As shown in Figure 6B we obtained a significant reduction in the LPS-enhancement of β-hexosaminidase release with the JNK inhibitor SP600125 (p<0.01). This result indicates that LPS-mediated enhancement of mast cell reactivity to IgE-receptor cross-linking occurs through the JNK dependent pathway.

## Discussion

The present investigation was directed to evaluate the impact of TRL engagement on mast cell reactivity to allergens. This study is the first of its kind to our knowledge which showed that prolonged treatment with TLR agonists, especially LPS, to CTLMC and MLMC, can induce augmented degranulation and release of CysLT and LTB_4_, as well as secretion of IL-6, MIP-1α, and MCP-1 following IgE-receptor cross-linking. Therefore in a clinical set up this observation would suggest that exposure to infections might exaggerate the severity of allergic reactions mediated by mast cells.

There is a considerable variation in the expression of TLRs among different species and subpopulations of mast cells. We therefore performed a PCR-array for TLR expression and found expression of all TLRs in both CTLMC and MLMC with especially high levels for TLR2, TLR4 and TLR6 (Fig. 1). This is in accordance with results of previous studies characterizing expression of TLR2, TLR4 and TLR6 in bone marrow derived mast cells (BMMC) [23], [24] and skin derived cultured mast cell [25]. Thus, in order to get an in depth quantitative and qualitative evaluation, we designed experiments with LPS for TLR4, which acts through both MyD88 dependent and independent pathways, FSL-1 and POC to discriminate between the TLR2 mediated MyD88 signals and also poly(I:C), which through TLR3 is the only receptor acting without MyD88 activation. We observed that none of the activated TLRs by themselves caused β-hexosaminidase release from either CTLMCs or MLMCs, neither after 24 h nor 96 h treatment. However, 96 h treatment with LPS, poly(I:C) and FSL-1 increased release of β-hexosaminidase from CTLMC following IgE receptor cross-linking (Fig. 2B). MLMC also showed increased β-hexosaminidase release following IgE-receptor activation but only after treatment with LPS (Fig. 2D). Interestingly, Supajatura et al. reported activation of mice BMMC after 1 h stimulation with a TLR2 agonist but not with a TLR4 agonist, indicating that under some conditions a short term effect of TLRs may alter mast cell response [13]. On the contrary, several other reports indicate that TLRs do not have any impact on mast cell degranulation upon simultaneous or short activation treatments [21], [24]. These observations support the absence of β-hexosaminidase release by TLR agonists or increased degranulation in combination with IgE-receptor cross-linking after 24 h treatment as we see in this study. However, there was a trend of augmentation in the IgE-induced β-hexosaminidase release after 24 h TLR agonist treatment. The gradual increase in response with time, as indicated in these experiments, is similar to the effect induced by LPS and poly(I:C) that have been observed earlier in mouse tracheal smooth muscle [26]. In those experiments, the up-regulation of the contractile responses to bradykinin in the tracheal segments obtained after 24 h treatment with both LPS and poly(I:C) was greatly increased after 96 h treatment. Thus, these results demonstrate that prolonged incubation by TLR agonists is needed to prime the mast cell for increased release activity.

In the present study IgE receptor cross-linking, but not TLR agonists alone, induced significant release of CysLTs and LTB_4_ from both CTLMC and MLMC. 96 h pre-treatment of both CTLMC and MLMC with TLR agonists further increased the IgE receptor mediated release of CysLTs and LTB_4_. LPS treatment enhanced IgE receptor cross-linking mediated release from both CTLMC and MLMC, while poly(I:C) and FSL-1 caused increased CysLTs release from MLMC and LTB_4_ release from CTLMC. This is in accordance with previous report demonstrating that poly(I:C) induced high release of CysLTs upon IgE-receptor cross-linking in human cultured mast cell [16]. These results further confirm the ability of TLR stimulation to modulate release of leukotrienes.

Secretion of IL-6, MIP-1α and MCP-1 after IgE-receptor cross-linking was markedly higher for MLMC than that of CTLMC. At the basal state MCP-1 release from CTLMC was significantly increased after TLR stimulation by LPS and poly(I:C). This differs from earlier studies on short term stimulation (from 20 min to 6 h) that showed IL-6, IL-13 and TNF-α release from mast cell line MC/9, peritoneum derived mast cells and BMMC [21], [24], [27]. Observed differences could be due to status of differentiation between the cell subtypes. Another possibility is that long-term treatment could have activated a feed-back regulation to down-regulate the cytokines, or that a different mechanism is involved in driving the secretion of MCP-1 [21], [24], [27]. Moreover, these studies show an effect of TLR2 and TLR4 both of which are mediated through the MyD88 pathway. We also show that the viral MyD88-independent pathway through TLR3 also induces release of MCP-1. However, when exploring the effect following IgE-activation only LPS induced a significant increase. This synergistic effect was seen for all three cytokines and chemokines in both cell subtypes and may be of specific importance in determining disease severity among asthmatic patients since these mediators both keep the inflammation on-going together with recruitment of inflammatory cells.

While investigating the mechanisms that induced the enhancing effect of TLRs, we could conclude, through experiments with MLMC from MyD88^−/−^ mice, that the LPS-induced increase of β-hexosaminidase release was mediated through MyD88-activation. However, since polyI:C also induced an increase of β-hexosaminidase release, this effect is not specific for MyD88. Furthermore, it can be speculated that the general more pronounced effect of LPS as compared to both the ligands that activate TLR2, which also mediated their effects through the MyD88 pathway, and poly(I:C), is because TLR4 stimulation activates both MyD88-dependent and independent signalling pathways [22]. Since neither of these TLR-induced effects was due to an increase in IgE expression (data not shown, we investigated the effectors in the MAPK pathway further down-stream of IgE receptor cross-linking and TLR-stimulation. Main effectors of these pathways involve MAPK signalling regulating activation of transcription factors such as NF-κB, c-Jun, c-Fos, ATF2, and NFAT, to induce cytokine-gene transcription. Here we detected phosphorylation of JNK, p38 MAPK and ERK1/2 only after IgE-receptor cross-linking, with no effect by TLR4 mediated activation. This suggests that the synergistic effect during IgE receptor cross-linking is separated from direct MAPK activation. When the JNK pathway was inhibited, during LPS treatment, a decrease in the extent of β-hexosaminidase release following IgE-receptor cross-linking was observed. These results indicate that during the 96 h treatment with LPS, JNK is activated and is involved in mediating the enhancement in degranulation upon IgE receptor cross-linking. A possible explanation for the LPS-mediated increase in mast cell reactivity was recently provided by Yang et al. [28]. They described that LPS-treatment of mast cells increase the expression of store operated calcium channels leading to increased calcium entry upon IgE-receptor mediated activation and concomitant increased mediator release.

Here we demonstrate that prolonged exposure of TLR agonists, specifically LPS in combination with IgE receptor cross-linking can enhance degranulation, secretion of leukotrienes and induce IL-6, MCP-1 and MIP-1α production from both CTLMCs and MLMCs; an affect which shorter incubation periods fail to achieve. This is the first study to elaborate the effector functions of CTLMCs and MLMCs after a prolonged TLR stimulus. Furthermore, our findings provide a mechanistic basis of how exposure to bacterial and viral infection might worsen the clinical adversities in allergic diseases as asthma.

## Materials and Methods

### Mast Cell Cultures

CTLMC and MLMC were derived by culturing mouse bone marrow cells from either wild type or MyD88^−/−^ C57BL/6 mice as previously described [29]. Animal experiments were approved by the regional committee of animal experimentation ethics, Stockholm North ethical committee for animal welfare, Stockholm, Sweden. In brief, CTLMCs were developed in medium containing recombinant murine stem cell factor (50 ng/ml; Peprotech, Stockholm, Sweden) and 1 ng/ml recombinant murine IL-4 (PeproTech). MLMCs were developed in medium containing IL-3 (5% supernatant of X63/0 myeloma cells transfected with an IL-3 expression vector), 5 ng/ml recombinant murine IL-9 (PeproTech), and 1 ng/ml recombinant human TGF-*β1* (PeproTech). All cells were cultured for a minimum of 2 weeks for MLMC and 4 weeks for CTLMC before they were used. Characterization and verification of *in vitro*-produced CTLMC and MLMC was performed through protease expression as previously described [29]. The maturity and purity of the cells were examined by toluidine blue staining and flow cytometry analysis for expression of Kit and FcεRI, using FITC-anti-mouse CD117 (Kit) mAb 2B8 or FITC-conjugated rat IgG2b isotype control (both from BD Pharmingen, San Diego, CA, U.S.A.), FITC-conjugated anti-mouse FcεRI-α mAb MAR-1, or FITC-conjugated Armenian hamster IgG isotype control (eBioscience, Frankfurt, Germany).

### Quantitative Real-time RT-PCR

Total RNA was extracted from CTLMC and MLMC cells according to manufacturer’s protocol (RNeasy mini kit, Qiagen, Sollentuna, Sweden). RNA was reverse-transcribed into single-stranded cDNA by using Quantitect reverse transcription kit (Qiagen). Quantitative real time RT-PCR was performed using Power SYBR Green PCR Master Mix (Applied Biosystems, Carlsbad, CA, USA) and TLR specific primers (Cybergene AB, Stockholm, Sweden; Table 1). The PCR reaction was optimized for each gene and 300 nM final concentrations of primers was assessed as the optimum for use over all conditions. A step down cycling protocol was used on the 7500 Real Time PCR instrument (Applied Biosystems). Hot start 95°C for 15 min followed by 66°C for 1 minute and 94°C for 30 seconds for 6 cycles with a decrease in temperature of 2°C for each cycle down to 56°C, followed by 40 cycles of 55°C and 94°C for 30 s. A melting curve was carried out at the end of the reactions. For the comparative QRT-PCR, the internal normalizer gene used was mouse β-actin. All mRNA expression data were normalized to these housekeeping genes and corrected using the reference dye (ROX) and performed in triplicates with intra-assay variations <0.5 where the mean value from each triplicate (>0.5 was excluded) were compiled together. Expression data was expressed in relation to Gmol β-actin as determined by software’s built in algorithm using an adaptive baseline to determine the cycle threshold (C_t_).

**Table 1:** Sequences of TLR and β-actin primer pairs.

Gene	Forward Primer 5′ to 3′	Reverse Primer 5′ to 3′
TLR1	gggaaaaagaagaccccgaa	gacacatccagaagaaaacggaa
TLR2	tgctcaggagtctctgtcatgtg	gcttttcatggctgctgtga
TLR3	tctgggctgaagtggacaaat	acctcaggcttgggagatagg
TLR4	agtggttgctgttcttattctgatttg	gacccatgaaattggcactca
TLR5	agccccgtgttggtaatatctc	tggtagtattgaggatccaggga
TLR6	gaatttggcaacctgacgaag	tgcagcttagatgcaagtgagc
TLR7	atccacaggctcacccatactt	agctagactgtttccttgaacatttg
TLR8	ctcacctaccctctggcttcc	gtttgcagggaggatttattgatc
TLR9	tgcaattggctgttcctgaa	ggtggtggatacggttggag
β-actin	tgggtcagaaggactcctatgtg	cgtcccagttggtaacaatgc

### Mast Cell Treatment and Activation

Before IgE-receptor activation, mast cells (1×10^6^ cells/ml), were treated 24 or 96 h with the TLR agonists LPS (1 µg/ml, Sigma-Aldrich, Stockholm, Sweden) [30], poly(I:C) (polyinosinic-polycytidylic acid;10 µg/ml, Sigma) [26], fibroblast stimulating lipopeptide (FSL-1; 100 nM, EMC Microcollection, Tübingen, Germany) and PamOct2C-(VPGVG)4VPGKG (POC; 100 nM, EMC Microcollection) [31]. In separate experiments to investigate the involvement of signalling pathway, mast cells were treated with the JNK inhibitor SP600125 (1 µM, Sigma), the ERK inhibitor PD 98059 (1 µM, Calbiochem, Stockholm, Sweden) and the p38 MAPK inhibitor SB203580 (10 µM, Calbiochem) in absence and presence with LPS for 96 h. After the TLR treatment the mast cells were washed, re-suspended in new medium, and activated through cross-linking of the IgE receptor. The cells were sensitised for 90 min with a monoclonal mouse anti-TNP IgE Ab (IgEl-b4; American Type Culture Collection) used as a 15% hybridoma supernatant followed by the addition of 50 ng/ml TNP-BSA (Biosearch Technologies, Novato, CA, USA) with a coupling ratio of 9∶1 for either 30 min for the measurement of β-hexosaminidase, CysLTs and LTB_4_ or 6 h (medium was supplemented with 10% FCS) to measure cytokine release.

### Measurement of Mast Cell Mediators: N-acetyl-β-D-hexosaminidase, Leukotrienes, Cytokines and Chemokines

For detection of the granular enzyme β-hexosaminidase, an enzymatic colorimetric assay was used as described previously [29]. Briefly, 60 µl of supernatant was transferred to a 96-well plate and mixed with an equal volume of substrate solution (7.5 mM *p*-nitrophenyl-*N*-acetyl-β-D-glucosaminide dissolved in 80 mM citric acid, pH 4.5). The mixture was incubated on a rocker platform for 2 h at 37°C. After incubation, 120 µl of glycine (0.2 M, pH 10.7) was added to each well, and the absorbance at 405 and 490 nm was measured. CysLT and LTB_4_ concentration were measured by a specific enzyme immunoassay with a commercial kit (Cayman Chemical Co, Ann Arbor, MI, USA). The immunoassay detection limit for CysLT and LTB_4_ were 7.8 pg/ml and 3.9 pg/ml, respectively. The concentrations of cytokines and chemokines in the culture supernatants were measured using mouse 10plex bead set (BioRad, Sundbyberg, Sweden) using the Luminex methodology. Labelled antibodies against the cytokines/chemokines, IL-1β, IL-6, IL-10, IL-13, IL-17, TNF-α, IFN-γ, MCP-1, MIP-1α, and GM-CSF were used for analyses of multiple cytokine responses according to manufacturer’s instructions. All measurements were performed in duplicates.

### Western Blot

To analyse MAPK activation, MLMC were cultured with medium alone or in the presence of LPS (1 µg/ml), activated by IgE-receptor cross-linking and analysed by Western blot. Cells were harvested 96 h after stimulation and lysed in SDS sample buffer (125 mM Tris-HCl, pH 6.8), 4% w/v SDS, 20% glycerol, 0.02% w/v bromphenol blue, and 50 mM DTT, added just before use) before sonication on ice. Western blotting was performed using NuPAGE Bis-Tris Western gels (Invitrogen, Stockholm, Sweden). Following electrophoresis, proteins were electroblotted onto nitrocellulose membranes (Hybond ECL; GE Healthcare, Uppsala, Sweden). Membranes were then blocked for 1 h in TBS containing 5% w/v nonfat dry milk and 0.1% Tween 20. Membranes were subsequently incubated overnight at 4°C with the primary antibodies rabbit anti-phosphorylated-ERK1/2 Ab, p JNK/SAPK antibody, p38 MAPK antibody (Cell Signaling Technology, Danvers, MA, USA), washed, and subsequently incubated with a HRP-conjugated secondary goat-anti rabbit Ab (Cell Signaling Technology) for 1 h at room temperature. The proteins were visualized using an ECL System LumiGLO and exposed to Hybond ECL film (GE Healthcare).

### Statistical Analysis

Results are expressed as mean ± SEM. Statistical analysis between two groups was performed by unpaired t-test and between multiple groups by one-way ANOVA followed by Newman-Keul’s post analysis test. Results were considered significant at p<0.05.
